# Ethical concern regarding “Magnetic resonance imaging features of bilateral multiloculated extraneural ganglion cysts of the occipito–atlanto–axial joint causing hypoglossal nerve paralysis in a dog”

**DOI:** 10.1093/jvimsj/aalag048

**Published:** 2026-03-25

**Authors:** Michael Brückner

**Affiliations:** Surgery Department, AWAKE Small Animal Hospital Helsingborg, Västra Sandgatan 7, 252 25, Helsingborg, Sweden

Dear Colleagues,

I am writing to you after reading the following article in your journal:

Magnetic resonance imaging features of bilateral multiloculated extraneural ganglion cysts of the occipito–atlanto–axial joint causing hypoglossal nerve paralysis in a dog by Minha Ji, Matti Kiupel, Hyungjin Park, Kichang Lee, and Hakyoung Yoon, DOI: http://dx.doi.org/10.1111/jvim.17192.

I would like to express my deep ethical concerns that this article was published in an internationally well-recognized peer-reviewed journal.

If it is true and the authors were not missing some information in their paper, then I do think that this article does not fully meet our ethical standards of care, since this patient underwent a major surgery without any analgesia, during and after the procedure, which is in my opinion unacceptable and should not have been published in the current version.

